# Socioeconomic Disparities in Individual-Level Quality-Adjusted Life Years throughout Remaining Lifetimes: A National Representative Longitudinal Survey in China

**DOI:** 10.3390/ijerph20054612

**Published:** 2023-03-05

**Authors:** Xinyi Huang, Xingtong Pei, Weiyan Jian, Mingming Xu

**Affiliations:** 1 School of Public Health (Shenzhen), Sun Yat-sen University, Gongchang Road 66, Shenzhen 518107, China; 2 Department of Health Policy and Management, School of Public Health, Peking University, Xueyuan Road 38, Haidian District, Beijing 100191, China

**Keywords:** socioeconomic status, QALYs, health disparities, survival analysis, China

## Abstract

Socioeconomic disparities in health within and across low- and middle-income countries pose a significant global public health concern. While prior research has demonstrated the importance of socioeconomic status on health outcomes, few studies have employed comprehensive measures of individual-level health such as quality-adjusted life years (QALYs) in exploring the quantitative relationship. In our study, we employed QALYs to measure individual-level health, using health-related quality of life scores based on the Short Form 36 and predicted remaining life years through individual-specific Weibull survival analysis. We then constructed a linear regression model to explore the socioeconomic factors that influence QALYs, providing a predictive model of individual-level QALYs throughout remaining lifetimes. This practical tool can help individuals predict their remaining healthy life years. Using data from the China Health and Retirement Longitudinal Study between 2011 and 2018, we found that education and occupation were the primary factors influencing health outcomes among individuals aged 45 and above, while income appeared to have less of an impact when education and occupation were simultaneously controlled for. To promote the health status of this population, low- and middle-income countries should prioritize the long-term advancement of their population’s education while controlling unemployment rates in the short term.

## 1. Introduction

It is widely recognized that health disparities exist between high-income countries and low- and middle-income countries (LMICs), both across and within LMICs [1,2,3]. For example, LMICs have higher cancer incidence and mortality rates [4], and being less wealthy, either as a country or as individuals, tends to be associated with poorer health outcomes, particularly among older people [3,5]. Socioeconomic status (SES) is an important social determinant of health and has received significant attention from academics and policymakers over the past few decades [2,4]. Currently, socioeconomic disparities in health within and between LMICs not only pose major global public health issues, such as poor health status and premature death among vulnerable populations [6], but also economic problems, including low economic efficiency. Poor health reduces productivity due to illness, further exacerbating socioeconomic status. The vicious cycle between poor health and low socioeconomic status reduces overall productivity and social stability, ultimately leading to lower economic productivity [7,8,9]. Therefore, a thorough understanding of socioeconomic disparities in health by clarifying the quantitative relationship between SES and health can help improve the health and well-being of middle-aged and elderly individuals, as well as achieve the goal of sustainable development [6,9].

Previous research has extensively explored the relationship between SES and health, including the underlying mechanisms and potential mediating factors [9,10,11,12,13,14,15,16,17,18,19,20,21,22]. Some researchers have found a positive relationship and explained the underlying mechanisms. At the individual level, SES impacts health status mainly through factors such as psychological stress [9,16,17,18,21,22]. At the group level, SES influences health by shaping the leading types of disease among certain subgroups, as demonstrated by the Whitehall study [11,12,23,24]. Others have focused on potential mediating factors. For example, subjective SES has been identified as an important mediating factor between objective SES and health [13]. Additionally, health literacy [14], lifestyle [15], environmental conditions, working conditions, and housing quality [19] have been considered as moderators of the effect of health on SES. Moreover, frailty, an age-related state of physical vulnerability, has been shown to influence human health through a negative relationship with SES as a mediating variable [20].

Regarding SES, different measurements are selected based on research purposes and study designs. Prior to 1985, researchers commonly used poverty status measured by income as an indicator before the emergence of the term SES [12]. However, since the Whitehall study [11], people have gradually realized the limitations of the old term and recognized that a person’s SES is determined not only by income [12,17,22] but also by other indicators such as occupation [12,13,15,17,18,25], education [12,13,17,18], public rental housing [16], area deprivation [18], and labor force group [26]. It is also worth noting that the SES model proposed by the WHO Commission on Social Determinants of Health in the report “Closing the gap in a generation” includes structural factors such as state power, income, distribution of goods and services, and daily living conditions such as access to healthcare, schools, and education among the social determinants affecting human health [9]. In conclusion, income, education, and occupation are the three most commonly adopted and recognized indicators of SES.

It is important to note that despite previous studies exploring the relationship between SES and both population-level and individual-level health, individual-level health has not been adequately measured. For population-level health, morbidity, mortality, and occasionally quality-adjusted life years (QALYs) have been used to measure the health status of a particular population and compare changes in health across gender or age groups [10,23,24,26]. QALYs present health as a time year, incorporating both the quantity and quality of life into a single measure, and are considered superior to other measures that only consider one aspect of health. When it comes to measuring individual-level health, several indicators have been commonly used, including self-perceived health status [27,28], the number of chronic diseases [10,11,18,29], limitations in activities of daily living [15], and health-related quality of life (HRQoL) [13,21,23,29,30,31]. However, these indicators fail to comprehensively reflect an individual’s true level of health. For instance, self-perceived health status is a subjective measure that heavily relies on an individual’s personal judgment and is influenced by factors such as education, income, and occupation [21,31]. Moreover, using self-rated measures as the dependent variable often reveals a stronger impact of income compared to education and occupation [13]. Therefore, QALYs, which provide a comprehensive and objective measure of health by incorporating both quantity and quality of life into a single metric, enable the correlation between an individual’s objective health status and SES to be analyzed, free from the interference of subjective factors.

In summary, while some previous studies have used QALYs to measure population-level health, comprehensive indicators of individual-level health such as QALYs are still scarce in the relevant literature. The main advantage of using QALYs to measure and compare individual-level health changes is its ability to comprehensively capture both the quality and quantity of life. However, this potential has not been fully utilized in studies examining the correlation between health and SES [22,32]. Simply measuring population-level health using QALYs makes it difficult to conduct empirical explorations and statistical analyses based on individual-level datasets [24,26]. Consequently, there remain limited empirical studies that quantify the relationship between SES and individual-level health using QALYs.

In this study, we aim to address this gap in the literature by measuring individual-level health using QALYs and quantifying the relationship between SES and health. As noted previously, individual-level QALYs, which combine quality and quantity of life, enable an empirical exploration of the correlation between SES and health using individual-level datasets [33] and facilitate the development of predictive models of QALYs in remaining lifetimes for individuals with specific characteristics. Therefore, our study aims to accomplish the following objectives: (1) generate individual-level QALYs by calculating HRQoL scores and fitting survival curves, (2) develop individual-specific predictive models of QALYs, and (3) investigate the relationship between SES and individual-level QALYs in remaining lifetimes. The results of this study will offer scientific evidence and quantitative tools to academics and policymakers alike who may utilize them to minimize population disparities and promote health in LMICs.

## 2. Methods

### 2.1. Data Source and Sample Selection

The China Health and Retirement Longitudinal Study (CHARLS) 2011, 2013, 2015, and 2018 were utilized for this study. CHARLS is a nationally representative longitudinal survey that collects data on Chinese middle-aged and older households aged 45 and older, including information on health status, socioeconomic status, and other relevant factors [34]. Only individuals enrolled in 2011 and followed up in at least one of the subsequent waves (2013, 2015, or 2018) were included in the study (n = 16,427). This is because only these individuals could contribute to survival analyses for estimating remaining lifetimes. Individual-level QALYs were obtained based on estimates of remaining lifetimes, utilizing the longitudinal dataset from 2011 to 2018. However, the linear regression was fitted only using the dataset from 2011, as the obtained QALYs indicated the remaining healthy life years in 2011.

### 2.2. Variables Specifications

In this study, we utilized *QALYs* as the dependent variable to measure an individual’s health status, which is considered to be strongly associated with HRQoL and remaining survival time based on the literature [27,28]. The *QALYs* of an individual, *i*, over their remaining lifetimes, *t*, can be computed as follows:

(1) $$QALYs_{i}=\int_{0}^{\infty }q_{i}\left(t\right)S_{i}\left(t\right)dt$$

where *q(t)* denotes the HRQoL score at time *t* and *S(t)* is the survival probability at time *t* [35]. The interval of the HRQoL score is [0, 1], with 0 and 1 denoting the state of death and perfect health, respectively. Specifically, a person with 0.8 HRQoL and 1 remaining life year would have 0.8 QALYs. In this paper, we obtain QALYs by multiplying the mean of HRQoL and the mean of *S(t)*, i.e., expected life years. 

The primary independent variables in this study are equivalent income (1st quartile: the poorest; 4th quartile: the richest), which is calculated by dividing household income by the square root of the household size [36]; education (1 = primary school or below, 2 = middle school, 3 = high school or above); and occupation (1 = agriculture work, 2 = employed, 3 = self-employed, 4 = retired or receded, 5 = unemployed). These variables together indicate an individual’s socio-economic status across three dimensions, according to the literature (note: “receded” refers to people who do not meet the retirement conditions but have completely lost their ability to work or have been approved to voluntarily leave their job positions) [11,14,16,18,19,29]. We also included age, gender (1 = male, 2 = female), marital status (1 = married/living together, 2 = single/living alone), and living areas (1 = urban, 2 = rural) as covariates.

### 2.3. Empirical Strategies

In this section, we describe the methods used to calculate HRQoL and survival probability before examining the association between QALYs and SES. HRQoL scores were obtained based on the Short Form 36 (SF-36) scale, a widely used measure of HRQoL [37]. Survival probabilities were estimated using the Weibull model, a parametric survival analysis method commonly used in health research [35,36,37,38,39,40]. Both analyses were conducted using Stata software for survival analysis and Origin software for function fitting. QALYs (unit: years) were calculated using Function (1). Lastly, we constructed a linear regression model to explore the relationship between QALYs and socioeconomic status variables (income, education, and occupation) and covariates (age, gender, marital status, and living areas).

#### 2.3.1. Short Form 36 (SF-36)

A widely used instrument, SF-36, was utilized to measure HRQoL [41,42]. It comprises 36 questions that assess general health, limitations of activities, emotional health, social activities, pain, and more. In this study, we calculated individual-level HRQoL based on selected questions from CHARLS that correspond to items in the SF-36 scale (refer to Supplementary Table S1 for details). Additionally, since HRQoL can be unpredictable, we estimated the HRQoL in the remaining lifetime by using the average score from 2011, 2013, 2015, and 2018, rather than a predicted score [35,38].

#### 2.3.2. Weibull Distribution

The survival probability is calculated using survival analysis techniques [7]. The survival curve is assumed to follow a Weibull distribution and is estimated as follows:

(2) $$\{\begin{array}{c} S\left(t_{i}\right)=e^{-\lambda_{i}t_{i}^{k}}\\ \lambda_{i}=e^{\alpha +X_{i}+\mu_{i}} \end{array}$$

where *t* denotes the time, *λ* and *k* are the two parameters of the Weibull distribution, *i* denotes an individual, *X* denotes covariates, and *μ* denotes the idiosyncratic error term. Among them, $\lambda_{i}$ is unique for each individual, enabling us to obtain unique QALYs for each individual. With the death information of the sample during 2011, 2013, 2015, and 2018, we can plot the survival curve of the population until 2018. As for the extended survival curve beyond 2018, we fit it by assuming a Weibull distribution. By extending the curve, we can obtain the longest possible lifespan at its junction with the x-axis. Accordingly, expected that life years for individual *i* could be estimated as follows, which is the mean of the Weibull distribution:

(3) $$\{\begin{array}{c} Lifeyears={\left(\frac{1}{\lambda_{i}}\right)}^{1/k}*\Gamma \left(1+1/k\right)\;\\ \Gamma \left(\zeta \right)=\int_{0}^{\infty }t^{\zeta -1}e^{-t}dt \end{array}$$

where Lifeyears denotes the expected life years, and Γ(ζ) is the gamma function. 

#### 2.3.3. Linear Regression

In order to explore the relationship between socioeconomic status and health status, we construct a linear regression model based on the sample from CHARLS 2011, shown as follows:

(4) $$\begin{array}{ll} ln\left(QALYs_{i}\right)= & \beta_{0}+\beta_{1}Income_{i}+\beta_{2}Education_{i}\\ \; & +\beta_{3}Occupation_{i}+\beta_{4}Age_{i}+\beta_{5}Ag{e_{i}}^{2}\\ \; & +\beta_{6}Gender_{i}+\beta_{7}Marital_{i}+\beta_{8}HRQoL_{i}\\ \; & +\beta_{9}Urban_{i}+u_{i} \end{array}$$

where *QALYs* is ln-transformed since it is non-negative. $\beta_{1}$, $\beta_{2}$, and $\beta_{3}$ are our focal estimates, denoting the correlation between the socioeconomic status and the dependent variable, i.e., $ln\left(QALYs_{i}\right)$. In addition, considering the potential collinearity between the variables, we conducted first univariate analyses before adding in the model all the other focal variables and covariates in the sequence.

## 3. Results

Based on the descriptive statistics presented in Table 1, our sample includes more than 16 thousand observations, with ages ranging from 45 to 101 years old. The average age of the sample is approximately 60 years old. On average, individuals in the sample have around 19 remaining years of life and 13.5 QALYs throughout their remaining lifetimes.

### 3.1. Individual-Level Life Expectancy

According to Functions (2) and (3), the estimated life expectancy for individual *i* can be calculated as:

$$\begin{array}{l} Lifeyears_{i}\\ =e^{-\left(0.10age_{i}-0.70gender\_female_{i}-0.43edu\_highschool_{i}+0.45occup\_unemployed_{i}+0.24marital\_single_{i}-2.89hrqol_{i}-9.31\right)/1.977}\\ {}*\;\Gamma \left(1.506\right). \end{array}$$

Based on the above results, individual-level life expectancies are related to demographic factors such as age, gender, and marital status, socioeconomic factors such as education attainment and occupation, and health factors such as HRQoL. Specifically, they are negatively correlated with age and positively correlated with HRQoL. Additionally, females and married individuals appear to have longer life expectancies. It is worth noting that among the main indicators of SES, individuals with education attainment of high school or above and those with non-unemployment status tend to have longer life expectancies than others, while income alone seems to have no significant influence on remaining life expectancy when education, occupation, and other covariates are controlled for.

To validate our individual-level life expectancy estimates in 2011, we compared the mean life expectancy by age obtained from our study with the publicly available data on World Life Expectancy in 2020 [43]. As shown in Supplementary Figure S1, the two curves exhibit a similar trend, indicating the plausibility of our estimates. As the data from the World Life Expectancy website are based on 2020 data, we adjusted it by subtracting two years since, according to the World Bank, life expectancy per Chinese citizen in 2020 is two years higher than in 2011 [44]. However, we acknowledge that this comparison is limited by the lack of direct evidence that the two-year difference represents the actual difference in average life expectancy between 2011 and 2020.

**Figure 1:**
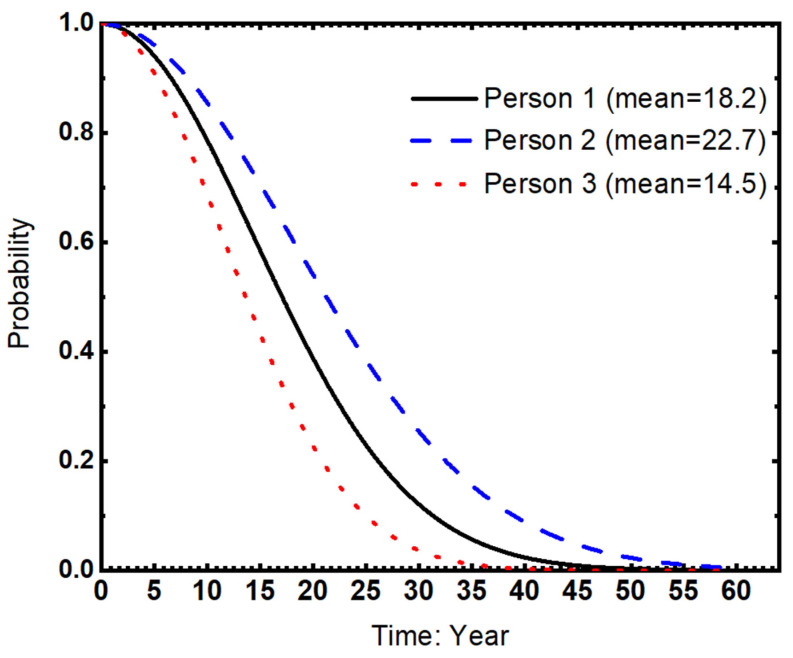
Individual-level life expectancy for simulated individuals. Note. Person 1: 60, male, primary school or below, agriculture work, married/living together, HRQoL = 1; Person 2: 60, male, high school or above, agriculture work, married/living together, HRQoL = 1; Person 3: 60, male, primary school or below, unemployed, married/living together, HRQoL = 1.

To further explore the association between life expectancy and socioeconomic status, we draw the survival curves for three simulated persons, who are assumed to have the same characteristics except for the socioeconomic statuses (details in the Note, Figure 1). If socioeconomic status has no or little impact on health, then the three curves representing different individuals will converge. As shown in Figure 1, the shapes of the survival curves for the three individuals are approximately the same by satisfying the Weibull distribution. As well, it is evident that Person 3 with the lowest socioeconomic status (primary school or below, and unemployed) has a lower life expectancy (14.5) than Person 2 (high school or above, and agriculture work) and Person 1 (primary school or below, and agriculture work) by approximately 8 and 4 years, respectively. 

To further investigate the association between SES and life expectancy, we plotted the survival curves for three simulated individuals with the same characteristics except for their SES (see details in the Note, Figure 1). If SES has little to no impact on health, then the three curves representing different individuals would be expected to converge. However, as shown in Figure 1, the survival curves for the three individuals follow the Weibull distribution and have distinct shapes. Notably, Person 3, with the lowest SES (primary school or below and unemployed) has a significantly lower life expectancy (14.5 years) compared to Person 2 (high school or above and working in agriculture) and Person 1 (primary school or below and working in agriculture) by approximately 8 and 4 years, respectively.

### 3.2. Individual-Level QALYs

The model for calculating individual-level *QALYs* can be expressed as:

$$\begin{array}{l} {\mathrm{QALYs}\;}_{i}\\ ={\mathrm{HRQoL}\;}_{i}\\ {}*\;e^{-\left(0.10age_{i}-0.70gender\_female_{i}-0.43edu\_highschool_{i}+0.45occup\_unemployed_{i}+0.24marital\_single_{i}-2.89hrqol_{i}-9.31\right)/1.977}\\ {}*\;\Gamma \left(1.506\right) \end{array}$$

Based on the above model, we can predict the QALYs for each individual with specific characteristics throughout their remaining lifetimes. QALYs take into account not only the length of life but also the quality of life, which intuitively would be expected to be lower than life expectancy. As depicted in Supplementary Figure S2, when comparing the mean individual-level life expectancy and the mean individual-level QALYs by age, a clear reduction in quality-adjusted life expectancy can be observed. Additionally, the rate of reduction increases with aging, indicating that the average quality of life declines as people age.

**Figure 2:**
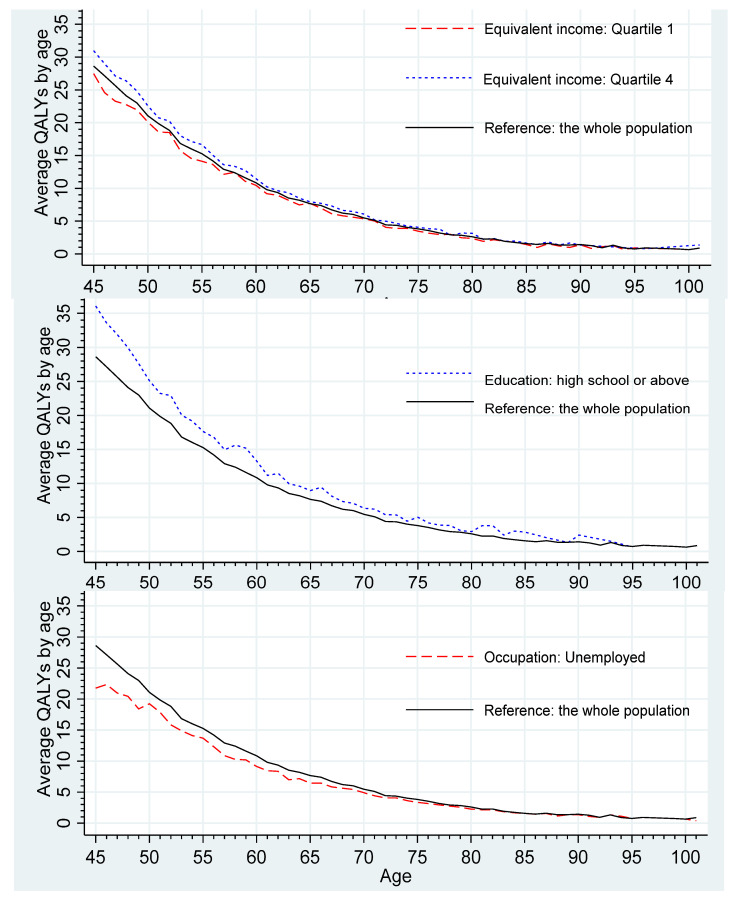
Average QALYs by age for the subgroups with varied socioeconomic statuses.

Figure 2 illustrates that individuals in the highest income quartile have higher QALYs across all age groups, while those in the lowest income quartile have lower QALYs. Moreover, compared to the overall population, individuals with a high school education or above and those who are employed tend to have higher QALYs, indicating longer quality-adjusted life years in the remaining lifetime. Notably, income appears to have a smaller impact on QALYs than education and occupation, as evidenced by the equivalent income lines being closer to the reference line.

### 3.3. The Impact of Socioeconomic Status on QALYs

In Table 2, we present the results of the impact of three indicators of SES on QALYs by adding focal variables and covariates in sequence. Detailed results can be found in Supplementary Table S2. Model 12 includes all relevant variables. The results of Model 12 show that people with an income above the median have significantly higher QALYs compared to the poorest people (quartile 1). Specifically, an increase in income from the first quartile to the third and fourth quartiles leads to a 0.79% ((EXP(0.0079) − 1) ∗ 100) and 0.88% ((EXP(0.0088) − 1) ∗ 100) increase in QALYs, respectively. While these results are significant, the absolute differences are small, indicating that income alone may not have a substantial impact on QALYs when other socioeconomic factors are held constant.

Regarding education, if individuals with an educational attainment of primary school or below can complete junior middle school or higher, their remaining QALYs can increase by 0.53% ((EXP(0.0053) − 1) ∗ 100) or 25.2% ((EXP(0.2245) − 1) ∗ 100), respectively. In terms of occupation, compared to those involved in agricultural work, the unemployed have 23.4% ((EXP(−0.2663) − 1) ∗ 100) shorter QALYs. Thus, income, education, and occupation, as crucial indicators of SES, have significant impacts on an individual’s QALYs, indicating that changes in SES are crucial for improving one’s health status.

The regression results, including focal variables and covariates added in sequence, are presented in Model 1–Model 10. Univariate analyses (Model 1–Model 6) revealed that the impacts of the three focal variables were exaggerated if relevant covariates were not controlled for. In addition, Model 7–Model 10 indicated that the three focal variables of SES indicators appeared to moderate each other, and the estimates became disturbed when the three indicators of SES were not simultaneously considered. The reason behind this is that any omitted variable that is correlated with a focal variable would be hidden in the idiosyncratic term, resulting in biased estimates of focal variables, and the collinearity problem could emerge. Therefore, to determine the true effects of SES indicators, we need to simultaneously control for income, education, occupation, and relevant covariates.

## 4. Conclusions

According to our findings, socio-economic status, particularly education and occupation, have a significant association with both life expectancy and QALYs. Income, on the other hand, is either less or not significantly related. Previous studies have shown that an increase in time spent in education reduces the risk of diseases and future mortality [45,46,47,48], promotes physical health, and increases life expectancy [47,48,49], which is consistent with our results. Our contribution to the literature is that we measure individual health using QALYs and present the quantitative relationship between education and health. Education influences health at the individual level by affecting personal characteristics. For example, individuals with higher educational attainment tend to adopt healthier lifestyles, which can directly benefit health [50,51]. Education also increases one’s capacity for knowledge and information, leading to better healthcare decision-making and utilization [52]. At the community level, education can influence health by improving physical and social environments. For instance, higher education reduces the likelihood of working in hazardous workplaces, lowering the risk of diseases and mortality [50].

Regarding occupation, only a few studies have shown a significant correlation between occupation and health [53], but our research adds to this evidence. Our findings indicate that unemployment is significantly negatively related to individual-level QALYs, with the QALYs of the unemployed being 23.4% shorter than those involved in agriculture work. Different occupation-specific workplaces can affect people’s working habits and exposure to risks differently, contributing to various health outcomes [50]. Moreover, our results are consistent with previous studies that suggest unemployment is a significant source of negative health outcomes, with epidemiological evidence showing that unemployment increases the risk of disease incidence and mortality [54]. One possible explanation for this is that unemployment can lead to an intensification of smoking and alcohol consumption, which in turn can increase the risk of physical illness, family breakdown, and psychological stress [55].

Our findings suggest that income is only weakly related to QALYs when controlling for education and occupation. However, previous studies have shown that income can have a stronger impact on health, particularly when income is below a certain threshold, such as the poverty line [11,18,28,56,57]. This suggests that income may only play a role in health at low levels. Additionally, income may act as an intermediary factor between education, occupation, and health, which could explain why income becomes less important when controlling for education and occupation. In other words, individuals with the same level of education and occupation but different incomes are likely to have similar health outcomes. This finding is consistent with other studies that have shown that education and occupation are equally as important as income for health outcomes [48].

In this study, we constructed a predictive model for individual-level QALYs throughout remaining lifetimes by exploring the quantitative relationship between socioeconomic status and health status, with individual-level measurements of health status created, namely QALYs. Although QALYs have been utilized in previous studies to measure health status, and survival analysis has been used to predict individuals’ remaining life years, these studies have mainly been at the population level, and the heterogeneity among individuals was often neglected [16,26,38]. Currently, few studies have quantitated health status at the individual level using QALYs, presumably due to the challenges of measuring individual survival probability and quality of life. However, with the help of HRQoL and individual-specific Weibull analysis, we were able to measure individual-level health by QALYs and construct the predictive model. Our model provides middle-aged or elderly individuals with a practical tool to predict their remaining healthy life years. Additionally, the model could be applied to other studies to examine health-related scientific questions and help with decision-making as an analytical tool.

Based on our findings that education and occupation are the two most significant socioeconomic factors for health, we can provide some policy implications. In order to address health disparities and improve people’s well-being in LMICs, it is crucial to prioritize education and reduce unemployment. In the short term, the government should make efforts to promote the economy and lower the unemployment rate, which is a fundamental factor for health, with income as an intermediate factor. Additionally, government regulation of the minimum wage is integral to guarantee the living conditions and health status of citizens. In the long term, the government should invest more in education to promote the average education level of the population, which is essential for steadily improving population health. These policies can help reduce health disparities and improve the overall well-being of the population.

It is important to acknowledge that our study has some limitations that should be taken into consideration. One of the main limitations is that the SF-36 was not fully utilized in calculating the HRQoL scores due to the limited relevant items in CHARLS, which might have resulted in a less precise measurement of HRQoL. However, previous studies have successfully addressed similar issues by mapping questionnaire items from other sources to the SF-36, as we did in our study [58,59]. Another limitation is that our analysis does not account for the influence of the COVID-19 pandemic in 2019, as the latest CHARLS database available is from 2018. Nevertheless, we believe that this pandemic may have negatively impacted the quality of life globally, and future studies should investigate this further. Lastly, it is important to note that our analysis has a relatively high level of aggregation, and caution should be exercised when applying our model to specific populations or individuals.

Last but not least, it is important to consider some ethical issues that may arise from our findings. While certain individuals may have higher QALYs, it is crucial for the government to allocate health resources in an equitable manner, without solely considering cost-effectiveness or prioritizing those who may gain more QALYs. Access to health resources should not be based on factors such as age, gender, and economic level, but rather on the basis of need and medical urgency, ensuring that everyone has equal opportunities to benefit from them. It is important to note that our research should not be used to support non-ethical policymaking, as this is not consistent with the purpose of our study. Rather, our aim is to help improve individuals’ QALYs and promote societal well-being.

## Figures and Tables

**Table 1:** Descriptive statistics in 2011.

Variables	The Whole Population		Middle-Aged (45–59)		The Elderly (≥60)	
	Obs	Mean/%	Obs	Mean/%	Obs	Mean/%
Age	16,427	59.5	9094	52.2	7333	68.4
Gender						
Female	8419	51.3%	4772	52.5%	3647	49.8%
Male	7996	48.7%	4315	47.5%	3681	50.2%
Occupation						
Agriculture work	6780	42.5%	3975	44.8%	2805	39.7%
Employed	2552	16.0%	2183	24.6%	369	5.2%
Self-employed	1228	7.7%	955	10.8%	273	3.9%
Retired or receded	480	3.0%	222	2.5%	258	3.7%
Unemployed	4909	30.8%	1547	17.4%	3362	47.6%
Education						
Primary school or below	4579	27.9%	1826	20.1%	2753	37.6%
Middle school	9836	60.0%	5758	63.4%	4078	55.7%
High school or above	1992	12.1%	1502	16.5%	490	6.7%
Equivalent income (CNY)	16,307	23,048	9034	24,413	7273	21,354
Marital status						
Married/living together	14,334	87.3%	8588	94.4%	5746	78.4%
Single/living alone	2093	12.7%	506	5.6%	1587	21.6%
Living area						
Urban	6323	38.4%	3542	39.0%	2780	37.9%
Rural	10,104	61.5%	5551	61.0%	4553	62.1%
Remaining life years	15,918	18.8	8868	26.0	7050	9.8
HRQoL	16,427	0.776	9094	0.715	7333	0.660
QALYs	15,918	13.5	8868	18.9	7050	6.7

**Table 2:** The impact of socioeconomic status on QALYs.

(1)						
INDEPENDENT VARIABLES	*ln(QALYs)*					
	Model 1	Model 2	Model 3	Model 4	Model 5	Model 6
Equivalent income: Quartile 1 (ref)						
Quartile 2	0.1217 *** (0.0171)	−0.0005 (0.0040)				
Quartile 3	0.3120 *** (0.0172)	0.0079 * (0.0041)				
Quartile 4	0.3265 *** (0.0173)	0.0086 ** (0.0043)				
Education:						
Primary school or below (ref)						
Middle school			0.3561 *** (0.0135)	−0.0113 *** (0.0034)		
High school or above			0.7776 *** (0.0201)	0.1859 *** (0.0051)		
Occupation:						
Agriculture work (ref)						
Employed					0.3752 *** (0.0154)	0.0276 *** (0.0037)
Self-employed					0.2781 *** (0.0206)	0.0062 (0.0047)
Retired or receded					−0.0416 (0.0313)	0.0399 *** (0.0071)
Unemployed					−0.7068 *** (0.0124)	−0.2430 *** (0.0032)
Covariates	No	Yes	No	Yes	No	Yes
Observations	15,809	15,809	15,918	15,918	15,918	15,918
**(2)**						
**INDEPENDENT VARIABLES**	***ln(QALYs)***					
	**Model 7**	**Model 8**	**Model 9**	**Model 10**	**Model 11**	**Model 12**
Equivalent income: Quartile 1 (ref)						
Quartile 2	0.0006 (0.0038)	−0.0004 (0.0038)	0.0827 *** (0.0147)	−0.0011 (0.0033)	0.0769 *** (0.0141)	0.0014 (0.0030)
Quartile 3	0.0029 (0.0039)	0.0009 (0.0039)	0.2508 *** (0.0150)	0.0105 *** (0.0034)	0.2090 *** (0.0144)	0.0079 *** (0.0031)
Quartile 4	−0.0112 *** (0.0040)	−0.0132 *** (0.0041)	0.3328 *** (0.0155)	0.0258 *** (0.0037)	0.2421 *** (0.0151)	0.0088 *** (0.0033)
Education:						
Primary school or below (ref)						
Middle school	0.3298 *** (0.0136)	−0.0103 *** (0.0034)			0.2404 *** (0.0117)	0.0053 ** (0.0027)
High school or above	0.7269 *** (0.0207)	0.1893 *** (0.0052)			0.6711 *** (0.0180)	0.2245 *** (0.0041)
Occupation:						
Agriculture work (ref)						
Employed			0.2702 *** (0.0158)	0.0216 *** (0.0038)	0.1524 *** (0.0155)	−0.0015 (0.0034)
Self-employed			0.1872 *** (0.0207)	0.0012 (0.0048)	0.1304 *** (0.0199)	−0.0019 (0.0043)
Retired or receded			−0.1606 *** (0.0314)	0.0323 *** (0.0072)	−0.3032 *** (0.0303)	−0.0057 (0.0065)
Unemployed			−0.7611 *** (0.0125)	−0.2475 *** (0.0032)	−0.7884 *** (0.0121)	−0.2663 *** (0.0029)
Covariates	No	Yes	No	Yes	No	Yes
Observations	15,809	15,809	15,809	15,809	15,809	15,809

Notes. The estimates stem from linear regression. Standard errors are in parentheses. Significance levels: * *p* < 0.1; ** *p* < 0.05; *** *p* < 0.01.

## Data Availability

Data available in a publicly accessible repository that does not issue DOIs. Publicly available datasets were analyzed in this study. This data (the China Health and Retirement Longitudinal Study, CHARLS) can be found here: [http://charls.pku.edu.cn/en/, accessed on 5 May 2022].

## Supplementary Materials

The following supporting information can be downloaded at: https://www.mdpi.com/article/10.3390/ijerph20054612/s1, Table S1: Questions, Table S2: The impact of socioeconomic status on QALYs, Figure S1: Life expectancy by age, Figure S2: Mean of individual-level QALYs and life expectancy by age.
