# Supplementary material for: Socioeconomic Disparities in Individual-Level Quality-Adjusted Life Years throughout Remaining Lifetimes: A National Representative Longitudinal Survey in China
Source: Int J Environ Res Public Health. 2023 Mar 5;20(5):4612. doi: 10.3390/ijerph20054612 (PMC10001792; doi:10.3390/ijerph20054612)
Supplement: Supplementary file 1 [file ijerph-20-04612-s001.zip › ijerph-2225188-supplementary.pdf]

Table S1. Questions

Short Form-36	CHARLS
**GENERAL HEALTH:**	
In general, would you say your health is:	**DA002** Next, I have some questions about your health. Would you say your health is very good, good, fair, poor or very poor?
Compared to one year ago, how would you rate your health in general now?	**DA002_W2_1** Compared with your health when we talked with you in R's LAST IW MONTH, YEAR, would you say that your health is better now, about the same, or worse?
**LIMITATIONS OF ACTIVITIES: The following items are about activities you might do during a typical day. Does your health now limit you in these activities? If so, how much?**	
Moderate activities, such as moving a table, pushing a vacuum cleaner, bowling, or playing golf.	**DB016** Because of health and memory problems, do you have any difficulties with doing household chores? (Definition: By doing household chores, we mean house cleaning, doing dishes, making the bed, and arranging the house)
Lifting or carrying groceries	**DB008** Do you have difficulty with lifting or carrying weights over 10 jin, like a heavy bag of groceries?
Climbing several flights of stairs	**DB005** Do you have difficulty with climbing several flights of stairs without resting?
Bending, kneeling, or stooping	**DB006** Do you have difficulty with stooping, kneeling, or crouching?
Walking more than a mile	**DB002** Do you have difficulty with walking 1 km?
Walking one block	**DB003** Do you have difficulty with walking 100 meters?

Bathing or dressing yourself	**DB010** Because of health and memory problems, do you have any difficulty with dressing? Dressing includes taking clothes out from a closet, putting them on, buttoning up, and fastening a belt.
	**DB011** Because of health and memory problems, do you have any difficulty with bathing or showering?
	**FC013** How many days of work did you miss in the past year due to health problems? 0…366 days
	**FD030** How many days of work did you miss at this current job in the past year due to health problems? 0…366 days
	**FH004** How many days of work did you miss in the past year due to health problems? 0…366 days
**PAIN:**	
How much bodily pain have you had during the past 4 weeks?	**DA041** Are you often troubled with any body pains?
**ENERGY AND EMOTIONS: These questions are about how you feel and how things have been with you during the last 4 weeks. For each question, please give the answer that comes closest to the way you have been feeling.**	
Did you feel full of pep?	**DC010** I had trouble keeping my mind on what I was doing. (<1 da； 1-2 days; 3-4 days; 5-7 days; DK; RF)

Have you felt so down in the dumps that nothing could cheer you up?	**DC011** I felt depressed. (<1 da； 1-2 days; 3-4 days; 5-7 days; DK; RF)
Have you been a happy person?	**DC016** I was happy. (<1 da； 1-2 days; 3-4 days; 5-7 days; DK; RF)
**GENERAL HEALTH:**<br>**How true or false is each of the following statements for you?**	
I expect my health to get worse.<br>(Definitely true; Mostly true; Don’t know; Mostly false; Definitely false)	**DA002_W2_1** Compared with your health when we talked with you in R’s LAST IW MONTH, YEAR, would you say that your health is better now, about the same, or worse?
My health is excellent.<br>(Definitely true; Mostly true; Don’t know; Mostly false; Definitely false)	**DA002** Next, I have some questions about your health. Would you say your health is very good, good, fair, poor or very poor?

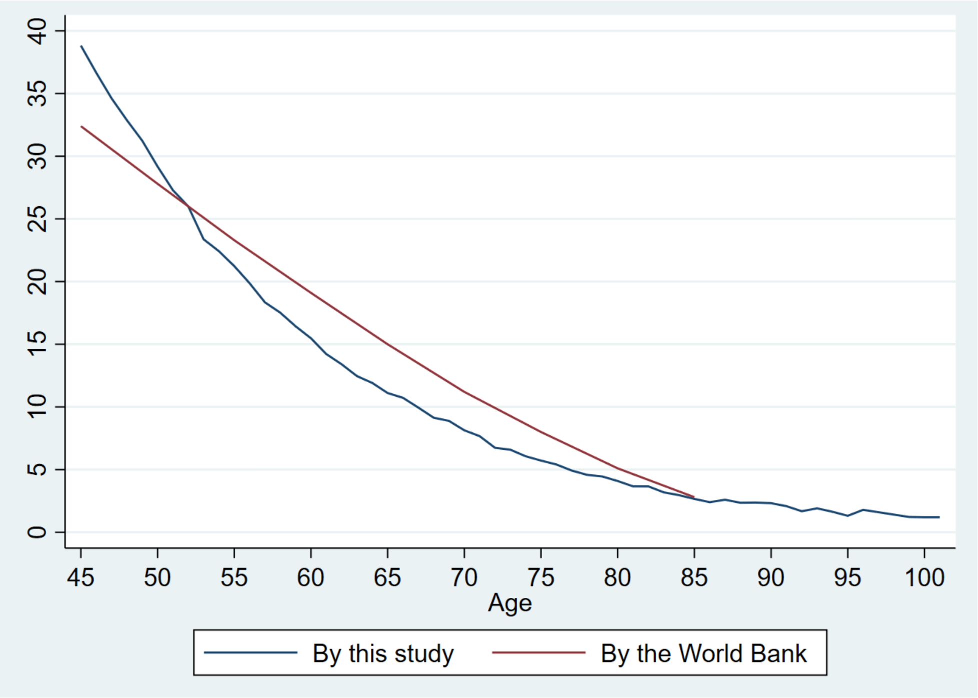

Figure S1. Life expectancy by age

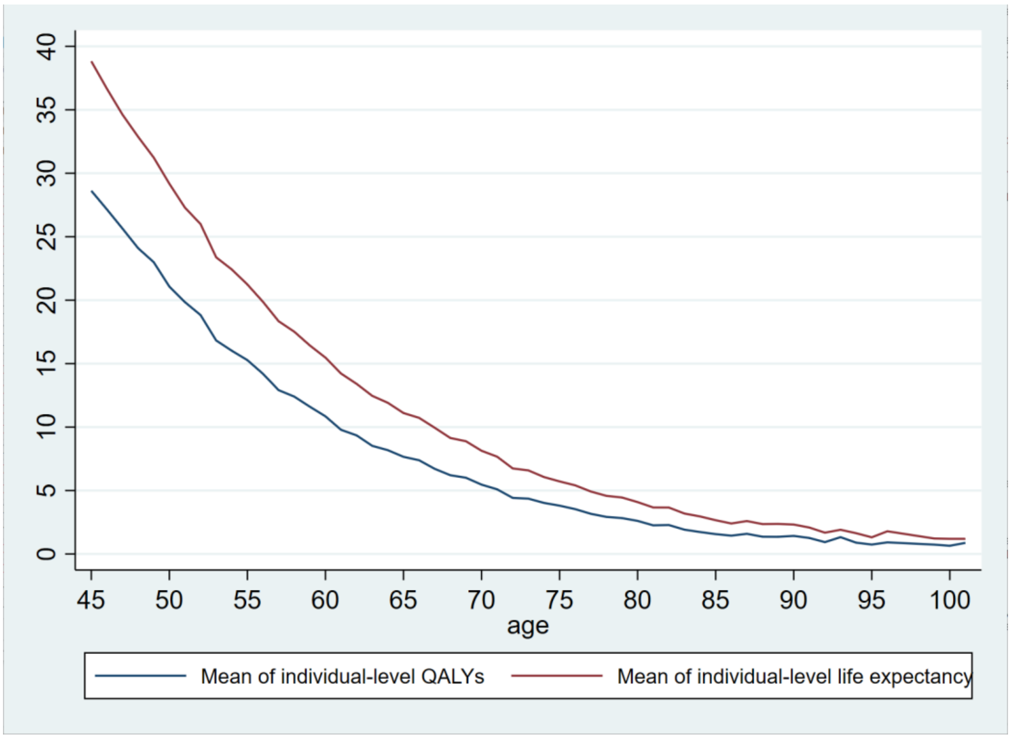

Figure S2. Mean of individual-level QALYs and life expectancy by age

Table S2. The impact of socioeconomic status on QALYs

(1)						
INDEPENDENT VARIABLES			*ln(QALYs)*			
	Model 1	Model 2	Model 3	Model 4	Model 5	Model 6
Equivalent income:						
Quartile 1 (ref)						
Quartile 2	**0.1217*** **	-0.0005				
	**(0.0171)**	(0.0040)				
Quartile 3	**0.3120*** **	**0.0079* **				
	**(0.0172)**	**(0.0041)**				
Quartile 4	**0.3265*** **	**0.0086** **				
	**(0.0173)**	**(0.0043)**				
Education:						
Primary school or below (ref)						
Middle school			**0.3561*** **	**-0.0113*** **		
			**(0.0135)**	**(0.0034)**		
High school or above			**0.7776*** **	**0.1859*** **		
			**(0.0201)**	**(0.0051)**		
Occupation:						
Agriculture work (ref)						
Employed					**0.3752*** **	**0.0276*** **
					**(0.0154)**	**(0.0037)**
Self-employed					**0.2781*** **	0.0062
					**(0.0206)**	(0.0047)
Retired or receded					-0.0416	**0.0399*** **
					(0.0313)	**(0.0071)**
Unemployed					**-0.7068*** **	**-0.2430*** **
					**(0.0124)**	**(0.0032)**
Age		-0.0473***		-0.0434***		-0.0486***
		(0.0015)		(0.0014)		(0.0013)
Age^2		-0.0001***		-0.0001***		-0.0000***
		(0.0000)		(0.0000)		(0.0000)
Gender:						
Male (ref)						
Female		0.3152***		0.3263***		0.3457***
		(0.0029)		(0.0029)		(0.0025)
Marital Status:						
Married/Living together						
Single/Living alone		-0.1493***		-0.1470***		-0.1339***
		(0.0047)		(0.0044)		(0.0039)
HRQoL		2.8641***		2.8134***		2.7278***
		(0.0149)		(0.0139)		(0.0123)
Living Areas:						

The urban (ref)						
The rural		-0.0334***		-0.0581***		0.0292***
		(0.0031)		(0.0029)		(0.0026)
Constant	2.1622***	3.3696***	2.0408***	3.2616***	2.4883***	3.4110***
	(0.0122)	(0.0488)	(0.0112)	(0.0456)	(0.0081)	(0.0408)
Observations	15,809	15,809	15,918	15,918	15,918	15,918
R-squared	0.0308	0.9472	0.0907	0.9535	0.2707	0.9637

(2)						
INDEPENDENT VARIABLES			*ln(QALYs)*			
	Model 7	Model 8	Model 9	Model 10	Model 11	Model 12
Equivalent income:						
Quartile 1 (ref)						
Quartile 2	0.0006	-0.0004	**0.0827*** **	-0.0011	**0.0769*** **	0.0014
	(0.0038)	(0.0038)	**(0.0147)**	(0.0033)	**(0.0141)**	(0.0030)
Quartile 3	0.0029	0.0009	**0.2508*** **	**0.0105*** **	**0.2090*** **	**0.0079*** **
	(0.0039)	(0.0039)	**(0.0150)**	**(0.0034)**	**(0.0144)**	**(0.0031)**
Quartile 4	**-0.0112*** **	**-0.0132*** **	**0.3328*** **	**0.0258*** **	**0.2421*** **	**0.0088*** **
	**(0.0040)**	**(0.0041)**	**(0.0155)**	**(0.0037)**	**(0.0151)**	**(0.0033)**
Education:						
Primary school or below (ref)						
Middle school	**0.3298*** **	**-0.0103*** **			**0.2404*** **	**0.0053** **
	**(0.0136)**	**(0.0034)**			**(0.0117)**	**(0.0027)**
High school or above	**0.7269*** **	**0.1893*** **			**0.6711*** **	**0.2245*** **
	**(0.0207)**	**(0.0052)**			**(0.0180)**	**(0.0041)**
Occupation:						
Agriculture work (ref)						
Employed			**0.2702*** **	**0.0216*** **	**0.1524*** **	-0.0015
			**(0.0158)**	**(0.0038)**	**(0.0155)**	(0.0034)
Self-employed			**0.1872*** **	0.0012	**0.1304*** **	-0.0019
			**(0.0207)**	(0.0048)	**(0.0199)**	(0.0043)
Retired or receded			**-0.1606*** **	**0.0323*** **	**-0.3032*** **	-0.0057
			**(0.0314)**	**(0.0072)**	**(0.0303)**	(0.0065)
Unemployed			**-0.7611*** **	**-0.2475*** **	**-0.7884*** **	**-0.2663*** **
			**(0.0125)**	**(0.0032)**	**(0.0121)**	**(0.0029)**
Age		-0.0435***		-0.0483***		-0.0448***
		(0.0014)		(0.0013)		(0.0011)
Age^2		-0.0001***		-0.0000***		-0.0001***
		(0.0000)		(0.0000)		(0.0000)
Gender:						
Male (ref)						

Female		0.3262***		0.3445***		0.3592***
		(0.0029)		(0.0025)		(0.0023)
Marital Status:						
Married/Living together						
Single/Living alone		-0.1483***		-0.1297***		-0.1258***
		(0.0044)		(0.0039)		(0.0035)
HRQoL		2.8183***		2.7167***		2.6605***
		(0.0140)		(0.0124)		(0.0111)
Living Areas:						
The urban (ref)						
The rural		-0.0562***		0.0252***		0.0041*
		(0.0030)		(0.0027)		(0.0024)
Constant	1.9333***	3.2618***	2.3669***	3.4027***	2.2096***	3.2931***
	(0.0143)	(0.0460)	(0.0113)	(0.0410)	(0.0129)	(0.0366)
Observations	15,809	15,809	15,809	15,809	15,809	15,809
R-squared	0.1050	0.9534	0.2959	0.9638	0.3530	0.9713

Notes. The estimates stem from linear regression. Standard errors are in parentheses. Significance levels: *p<0.1; **p<0.05; ***p<0.01.
